# Melitid amphipods from the Gulf of Thailand, with a description of *Dulichiella pattaniensis*, a new species

**DOI:** 10.3897/zookeys.408.7292

**Published:** 2014-05-10

**Authors:** Koraon Wongkamhaeng, Manasawan Saengsakda Pattaratumrong, Ratchanee Puttapreecha

**Affiliations:** 1 Marine and Coastal Resources Institute (MACORIN), Prince of Songkla University, 90112 Thailand; 2 Southern Marine and Coastal Resources Research Center,158 Moo 8, Phawong, Muang, Songkhla 90100 Thailand

**Keywords:** Crustacea, Amphipoda, Melitidae, *Dulichiella pattaniensis*, Gulf of Thailand, taxonomy

## Abstract

Two species of melitid amphipod were collected from the Gulf of Thailand. *Dulichiella pattaniensis* is new to science, and *Melitalati latiflagella* Ren & Andres, 2012 has not been previously reported from Thai Waters. *Dulichiella pattaniensis* is characterized by male gnathopod 2 distolateral crown with 4 spines; pleonite/urosomite formula 7-7-7-5-6-2; pereopod 5-7 dactylus with 2 accessory spines. This combination of characters has not been recorded previously in the *Dulichiella*. The characters of the specimens are described and illustrated. All specimens are deposited in the Princess Maha Chakri Sirindhorn Natural History Museum, Prince of Songkla University, Thailand.

## Introduction

Melitid amphipods most commonly occur in coastal and freshwater areas. Thailand has a variety of aqueous habitats including coral reefs, seagrass beds, and mangrove forests, but only one melitid amphipod, *Rotomelita longipropoda* Wongkamhaeng et al., 2013 was described. In this study, we describe a new melitid species *Dulichiella pattaniensis* sp. n., and our observations of *Melita latiflagella* Ren & Andress, 2012, which has not been previously reported in Thai Waters. Figures and descriptions of both species are provided.

## Materials and methods

**Figure 1.**
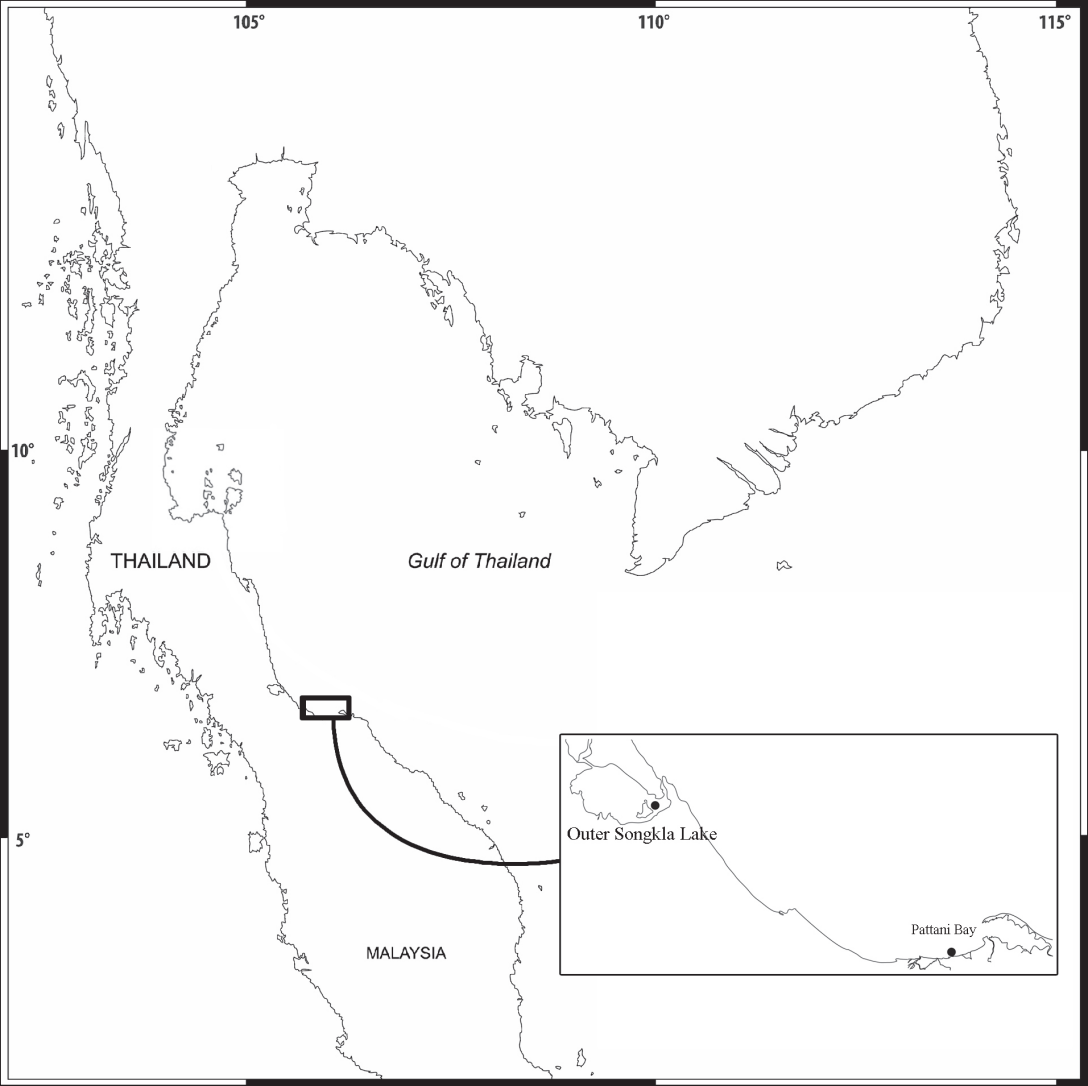
Map of the sampling area.

Amphipods were collected from some settlement plates in an artificial reef in Ban Pak Bang Ta Wa, Pattani Bay and from sediment of Lower Songkhla Lake (Figure 1). The sites were visited at low tide and amphipods were collected using a 20×20 cm Ekman grab from the subtidal zone. The amphipod specimens were sorted out and fixed in formalin for 1 week and then stored in 70% alcohol. In the laboratory, the specimens were transferred from alcohol into glycerol for study. Drawing was accomplished using a drawing tube attached to an Olympus CH30 light microscope. The pencil drawings were scanned and digitally inked using a WACOM bamboo CTH-970 graphics board following the method described in Coleman (2003). The following abbreviations are used: A, antenna; G, gnathopod; HD, head; LL, lower lip; MD, mandible; MX, maxilla; MP, maxilliped; P, pereopod; Pl, pleopod; T, telson; U, uropod; UR, urosome; UL, upper lip; r, right; l, left; ♂, male; ♀, female. Specimens of different species were deposited into the Prince of Songkla University Zoological Collection (PSUZC).

## Results

### Systematics
Melitidae Bousfield, 1973

#### Dulichiella

Stout, 1912

http://species-id.net/wiki/Dulichiella

**Figure 2.**
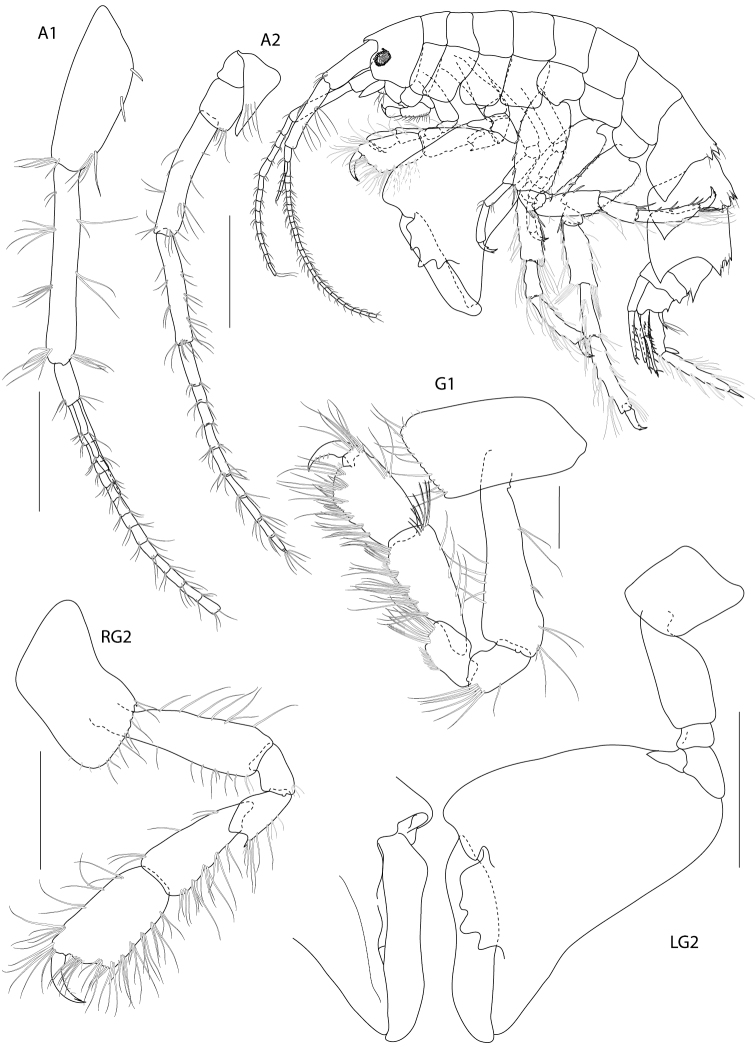
*Dulichiella pattaniensis* sp. n. holotype, male, (PSUZC-CR-00192), 6.3 mm. Pattani Bay, Lower Gulf of Thailand. All scale bars represent 0.5 mm.

Figures 2 [Fig F3] [Fig F4] [Fig F5] [Fig F6] [Fig F7] [Fig F8] –9

##### Diagnosis.

(Lowry and Springthorpe 2007) Head anteroventral corner with several long, slender setae. Antenna 1 longer than antenna 2. Maxilla 1 inner plate long, narrow, tapering distally, with 2 well developed apical plumose setae. Maxilla 2 inner plate with oblique setal row. Gnathopod 2 male, asymmetrical, significantly unequal in size; palm in larger slightly obtuse; those of female equal in size. Pereopods 5–7 distal articles strongly to weakly setose; dactylar ungues with accessory spines. Pereopods 6 and 7 in males with bunches of long slender setae. Pereopod 7 basis in female fully expanded. Pleonites dorsally serrate. Uropod 3 inner ramus scale-like; outer ramus 4 to 5× longer than wide, 2-articulate. Telson deeply cleft, lobes tapering distally to an acute point.

##### Typespecies.

*Dulichiella spinosa* Stout, 1912 (type by monotypy).

##### Species composition.

*Dulichiella appendiculata* Say, 1818; *Dulichiella australis* Haswell, 1879; *Dulichiella celestun* Paz-Rios & Ardisson, 2014; *Dulichiella cotesi* Giles, 1890; *Dulichiella cuvettensis* Appadoo & Myers, 2005; *Dulichiella fresnellii* (Audouin, 1826); *Dulichiella guinea* Lowry & Springthorpe, 2007; *Dulichiella lecroyae* Lowry & Springthorpe, 2007; *Dulichiella oahu* Lowry & Springthorpe, 2007; *Dulichiella pacifica* Lowry & Springthorpe, 2005; *Dulichiella pattaniensis* sp. n.; *Dulichiella spinosa* Stout, 1912 (type species); *Dulichiella takedai* Tomikawa & Komatsu; *Dulichiella terminos* Lowry & Springthorpe, 2007; *Dulichiella tomioka* Lowry & Springthorpe, 2007; *Dulichiella tulear* Lowry & Springthorpe, 2007.

**Figure 3. F3:**
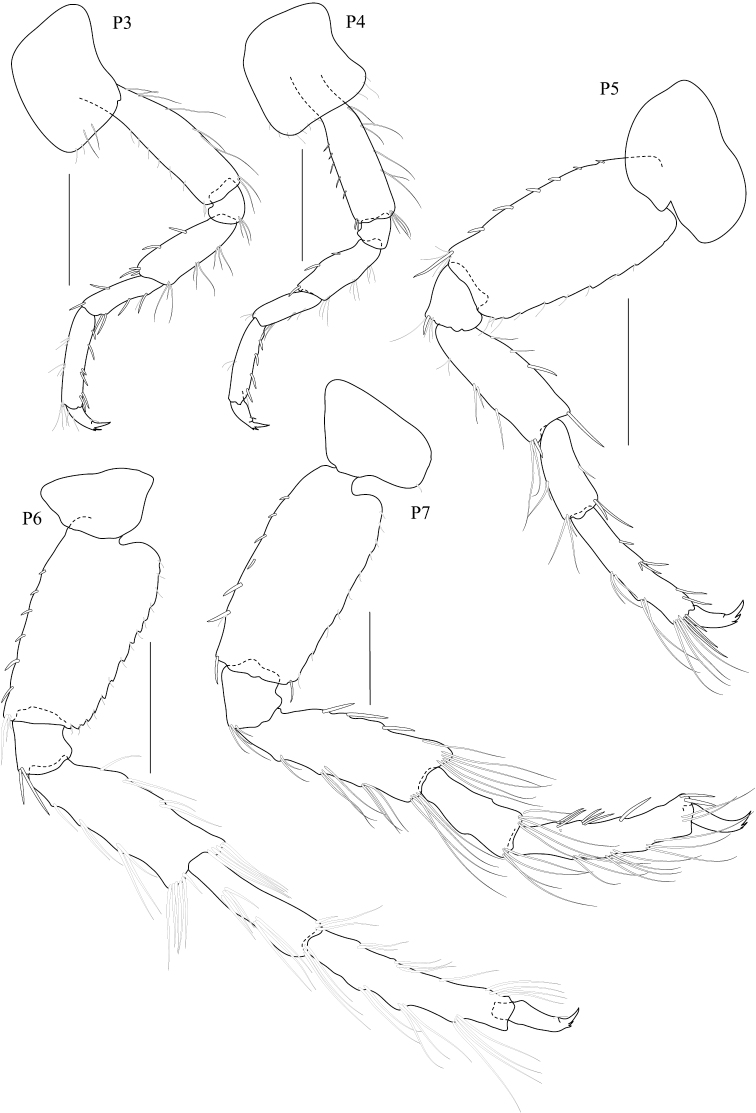
*Dulichiella pattaniensis* sp. n. paratype, male, (PSUZC-CR-00194), Pattani Bay, Lower Gulf of Thailand. All scale bars represent 0.5 mm.

**Figure 4. F4:**
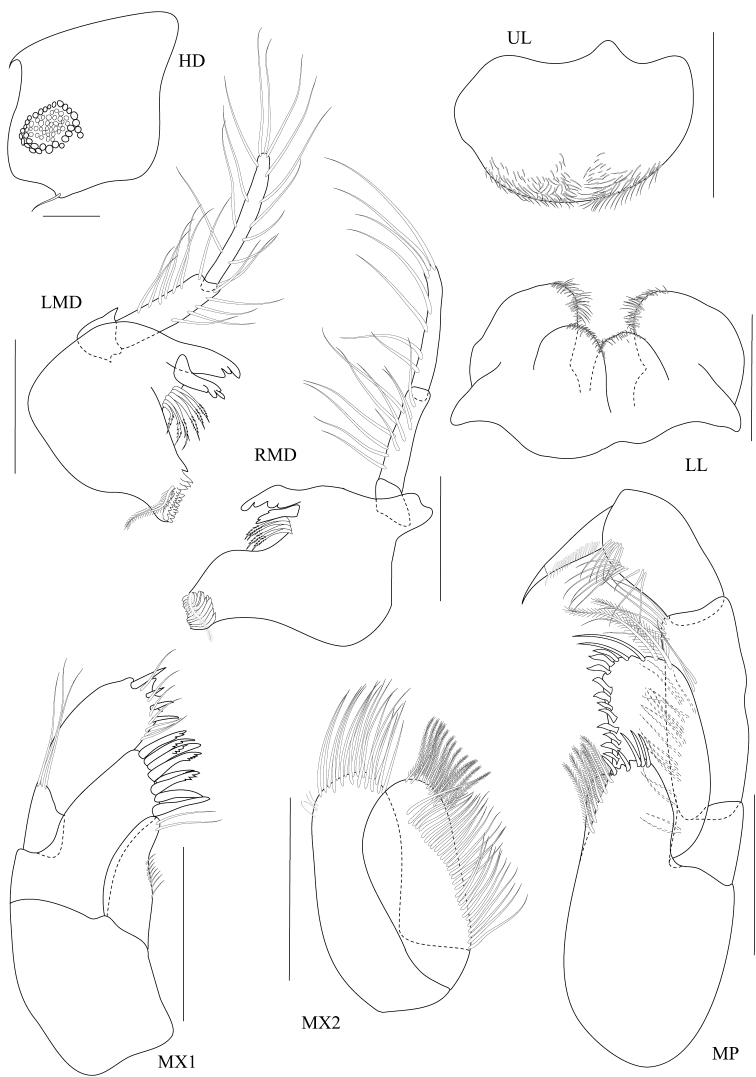
*Dulichiella pattaniensis* sp. n. paratype, male, (PSUZC-CR-00194), Pattani Bay, Lower Gulf of Thailand. The scale bars for U1-U3, PL1-3 represent 0.5 mm, but 0.2 mm for T.

**Figure 5. F5:**
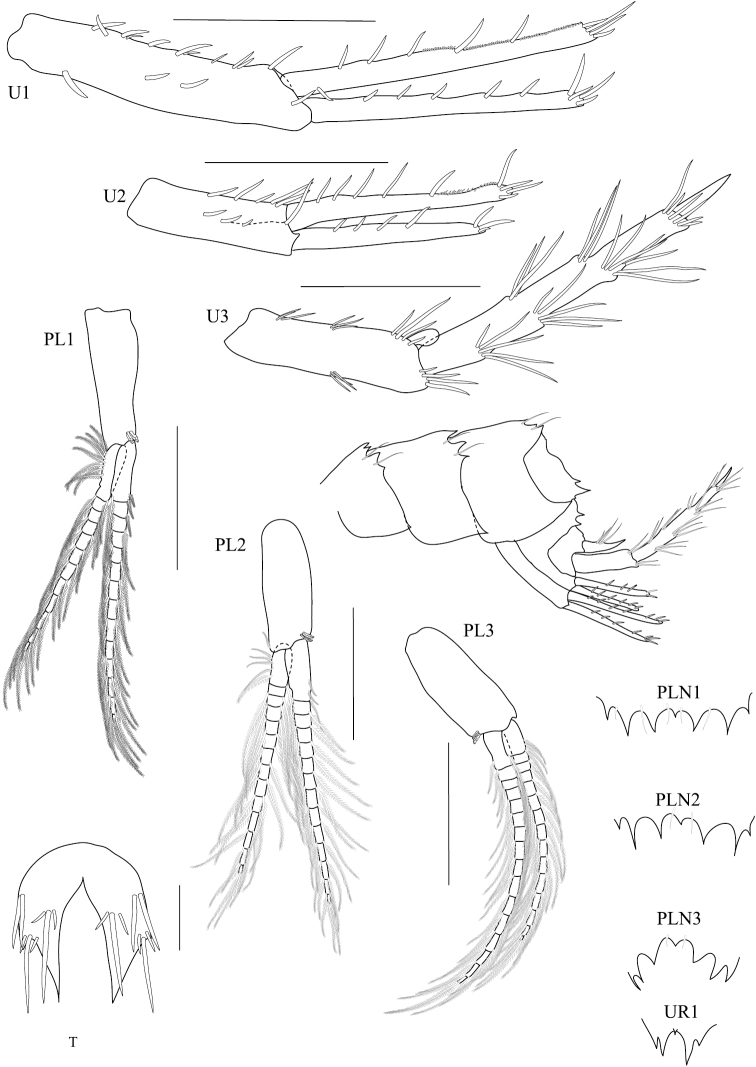
*Dulichiella pattaniensis* sp. n. paratype, male, (PSUZC-CR-00194), Pattani Bay, Lower Gulf of Thailand. All scale bars represent 0.5 mm.

**Figure 6. F6:**
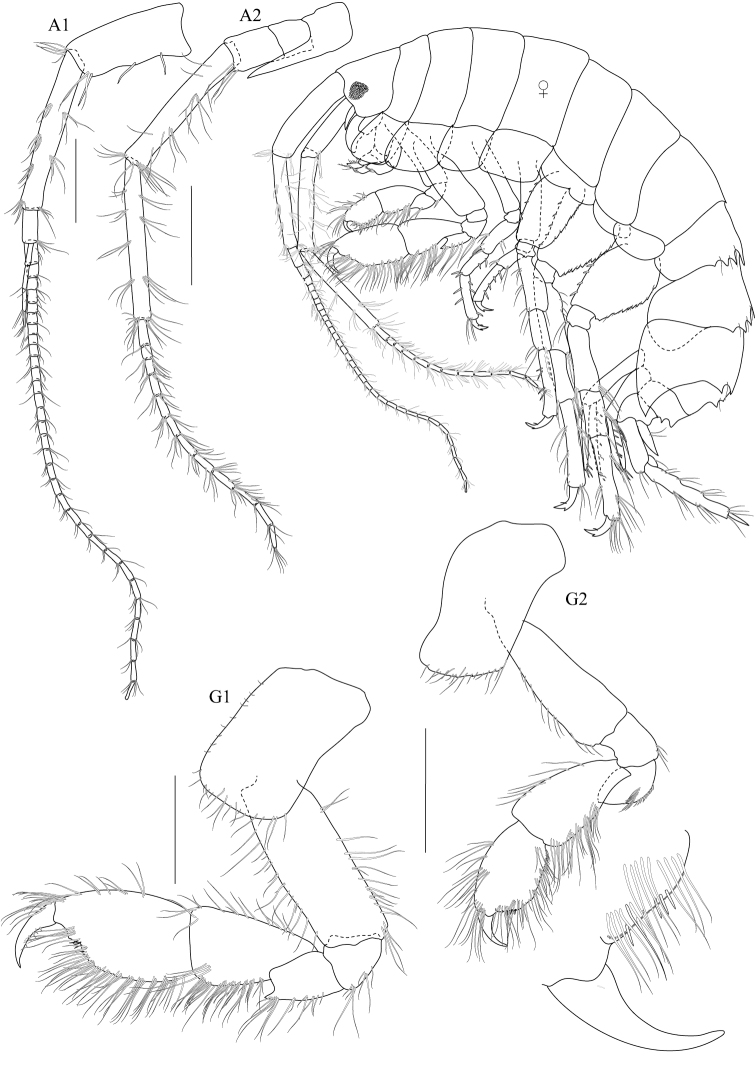
*Dulichiella pattaniensis* sp. n. allotype, female, (PSUZC-CR-00193), Pattani Bay, Lower Gulf of Thailand. All scale bars represent 0.5 mm.

**Figure 7. F7:**
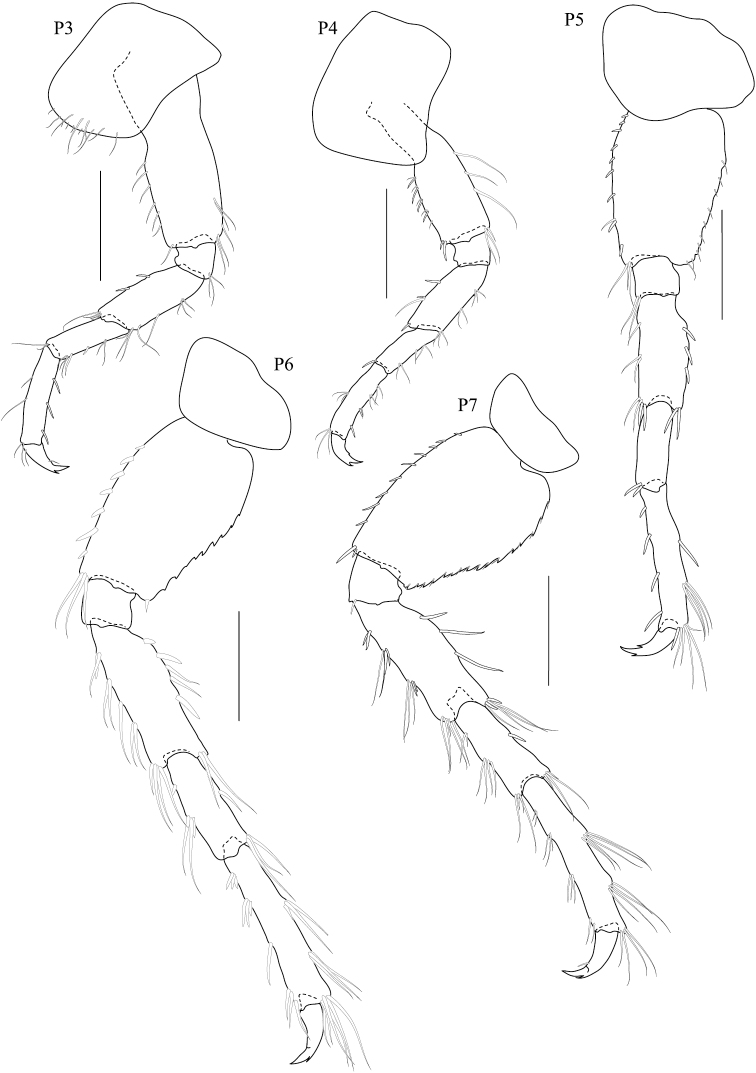
*Dulichiella pattaniensis* sp. n. allotype, female, (PSUZC-CR-00193), Pattani Bay, Lower Gulf of Thailand. All scale bars represent 0.5 mm.

**Figure 8. F8:**
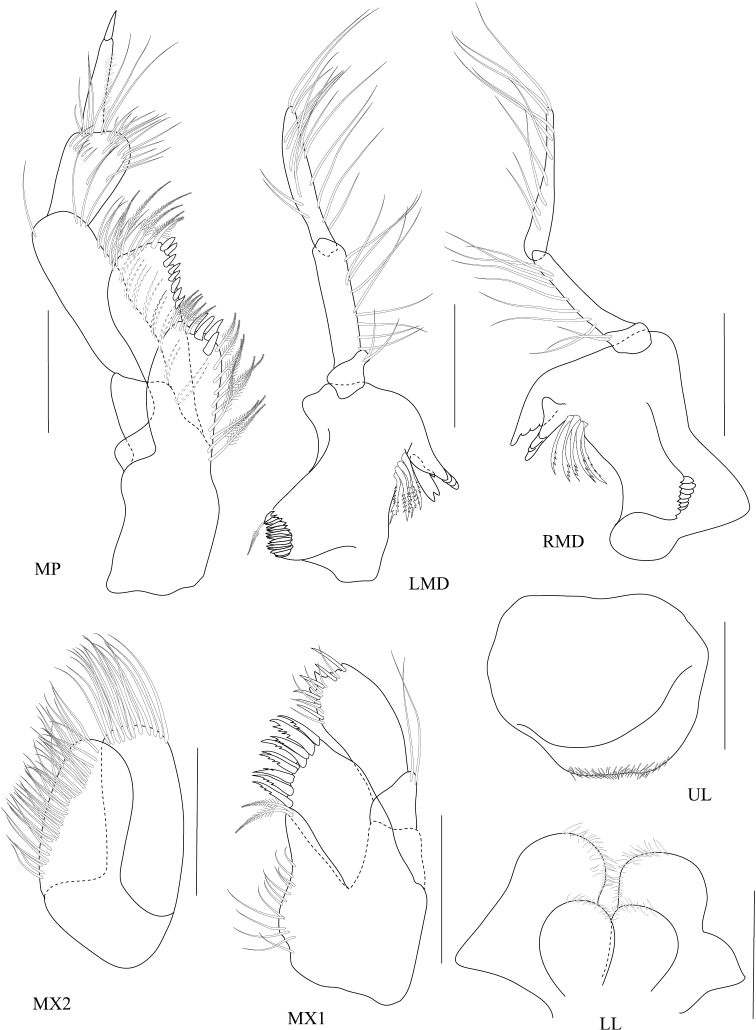
*Dulichiella pattaniensis* sp. n. allotype, female, (PSUZC-CR-00193), Pattani Bay, Lower Gulf of Thailand. All scale bars represent 0.2 mm.

**Figure 9.**
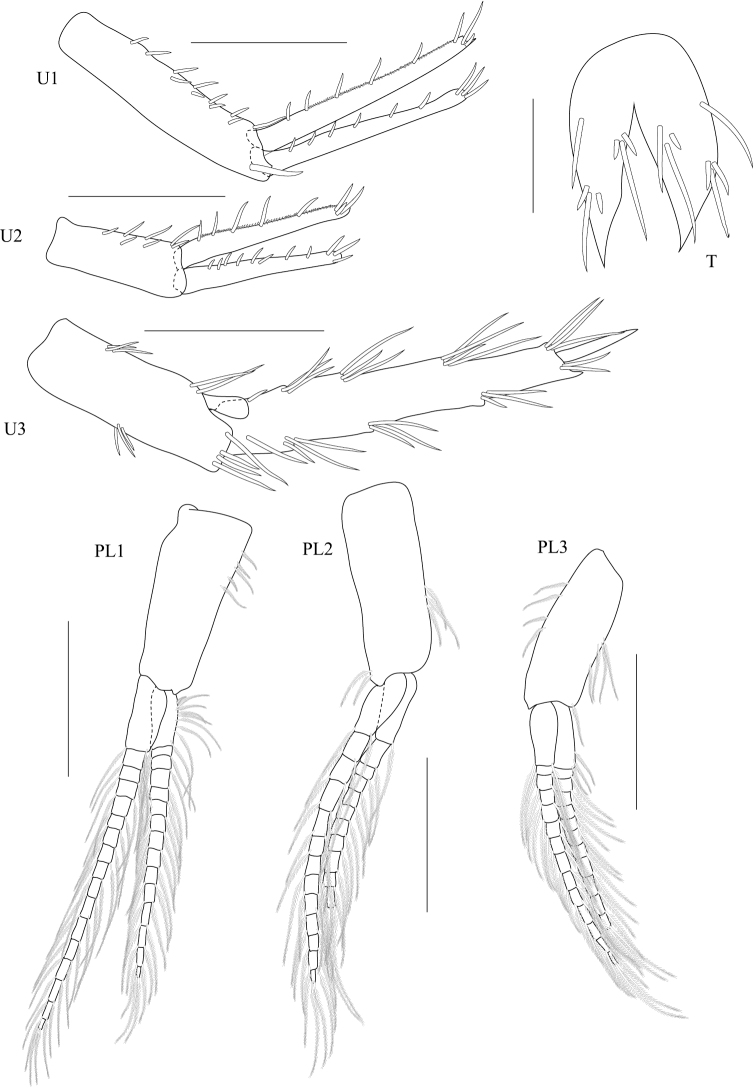
*Dulichiella pattaniensis* sp. n. allotype, female, (PSUZC-CR-00193), Pattani Bay, Lower Gulf of Thailand. Scale bars for U1-U3, PL1-3 represent 0.5 mm, but 0.2 mm for T.

#### Dulichiella pattaniensis sp. n.

http://zoobank.org/212F51D8-E32A-4601-9E04-88292FBEB989

http://species-id.net/wiki/Dulichiella_pattaniensis

##### Type material.

Holotype. ♂, THAILAND, Lower Gulf of Thailand, Pattani Bay (6°51'55"N, 101°10'7"E), artificial reef (associated with coral settlement plate), 1 September 2010, Puttapreecha, R., PSUZC-CR-0192. Allotypes, ♀ collected with holotype; PSUZC-CR-0193; Paratype, collected with holotype (PSUZC-CR-0194 (5♂; 5♀)).

##### Description.

Based on male holotype. Body length 6.3 mm (from tip of rostrum to apex of telson). *Body* compressed, subcylindrical. *Head*, lateral cephalic lobe truncate, anteroventral corner with setae, eyes round. *Antenna 1*, setiferous, ratios of peduncular articles 1–3 4:5:1; peduncular article 1 with 3 ventromarginal robust setea and distoventral setae; accessory flagellum with 5 articles, last article reduced; primary flagellum 16-articulate (possibly regenerating in this specimen), flagellum can be 25-articulate (observed from additional material). *Antenna 2*, Antenna 2 setiferous, peduncular article 2 cone gland not reaching end of article 3; article 5 subequal to 4, flagellum 9-articulate.

*Upper lip*, (labrum) distally rounded. *Lower lip*, inner lobes well developed, pubescent. *Mandible*, both similar, left incisor 3 dentates, right incisor 4 dentate; left and right lacinia mobilis armed with 3 and 4 dentates respectively; palp slender with marginal setae, article 1 smooth, article 3 slightly longer than article 2. *Maxilla 1*, inner plate narrow with 2 apical plumose setae; outer plate with 8 apical serrate robust setae; palp 2-articulate, article 1 with 3 distal setae, article 2 with 4 apical robust setae and 4 apical setae. *Maxilla 2*, inner plate with mediofacial row of 29 setae and 14 apical plumose setae; outer plate broader than inner plate, distally setose. *Maxilliped*, inner plate borad, with 6 plumose marginal setae; outer plate margin with 11 conate robust setae, terminal with 4 plumose setae; palp 4-articulate, article 2-3 with marginal setae, article 4 tapering with fine marginal setae.

**Pereon.** *Gnathopod 1* subchelate, smaller than gnathopod 2; coxa anterodistal corner not produced, posteroventral corner notch present, anterior margin straight; length ratio of articles from basis to dactylus 10: 3:4:7:5: 3; basis slender; merus–propodus setose; palm slightly convex, defined by posterodistal corner, without posterodistal robust setae. *Gnathopod 2* sexually dimorphic; left and right gnathopods unequal in size; coxa posteroventral corner notch present; (larger) length ratio of article from basis to dactylus 8:1:3:1:10:11; propodus distolateral corner crown with 4 rounded spines, palm straight, posterodistal corner produced, upturned, fit with dactylus; dactylus apically blunt; (smaller) subchelate; length ratio of article from basis to dactylus 9:3:4:6:6:4; merus with sharp posteroventral spine; carpus subequal to propodus; palm straight, without posteroventral spine. *Pereopod 3–4* alike. *Pereopod 5* basis posterior margin straight, posteroventral corner rounded; carpus and propodus sparsely setose; dactylus unguis anterior margin with accessory spines. *Pereopod 6–7* alike, basis, merus, carpus, propodus with long marginal setae. *Pereopod 6* basis posterior margin straight, minutely castelloserrate; dactylus unguis anterior margin with accessory spines. *Pereopod 7* basis posterior margin straight, with posterior margin minutely castelloserrate, posteroventral corner; dactylus unguis anterior margin with accessory spines.

**Pleon.** Pleonite/urosomite dorsal spine formula (7-7-7-5-6-2). *Pleonites 1–3* with dorsal setae. *Epimera 1–3* posteroventral margin without spines above posteroventral corner. *Epimeron 3* posterior margin smooth, posteroventral corner with strongly produced acute. *Urosomite 1* with spine at midline, no medial gape. *Urosomite 2* with dorsal setae. *Urosomite 3* with dorsal setae, with 2 dorsal spines. *Uropod 3* inner ramus scale-like, much shorter than outer ramus; outer ramus much longer (more than 2× length) than peduncle, 2-articulate. *Telson* with dorsal robust setae.

**Female. (sexually dimorphic characters).** Length, 7.4 mm. Gnathopod 1 coxa 4 anterodistal corner not produced, posteroventral corner notch present, anterior margin excavated. Gnathopod 2 equal, coxa subrectangular, palm crenulated, oblique with setae on margins. Pereopod 7 basis expanded, posterior margin slightly convex.

##### Etymology.

This species is named after the type locality.

##### Remarks.

*Dulichiella pattaniensis* sp. n., with pleonite/urosome formular of 7-7-7-5-6-2 has only *Dulichiella cotesi*, *Dulichiella oahu*, *Dulichiella pacifica* and *Dulichiella tulear* that share this characters. This new species can be distinguished from *Dulichiella cotesi*, *Dulichiella oahu* and *Dulichiella tulear* by having male gnathopod 2 (large) with 4 spines on distolateral crown while those three species have 3 spines. *Dulichiella pattaniensis* differs from *Dulichiella pacifica* in the following: the male large gnathopod 2 has a fourth spine on its distolateral crown that is not well developed vs. its well-developed fourth set of spines. The male gnathopod 1 has carpus longer than its propodus vs. a carpus subequal to its propodus. Pereopods 3-4 have a dactyli with 2 accessory spines vs. 3-4 dactyli with 1 accessory spine.

The new species also has four spines on the distolateral crown of the male gnathopod 2. Only 7 species, *Dulichiella appendiculata*, *Dulichiella cuvettensis*, *Dulichiella celestun*, *Dulichiella fresnellii*, *Dulichiella guinea*, *Dulichiella lecroyae*, *Dulichiella pacifica* and *Dulichiella takedai* share this distinct character. *Dulichiella pattaniensis* can be distinguished from amphipods by having pleonite/urosome formular of 7-7-7-5-6-2 while *Dulichiella appendiculata*, *Dulichiella cuvettensis*, *Dulichiella fresnellii*, *Dulichiella lecroye* and *Dulichiella pacifica* pleonite/urosome formula 7-7-7-5-4-2, *Dulichiella guinea* 9-9-7-5-4-2 and *Dulichiella celestun* 9-9-9-5-6-2. Moreover, *Dulichiella pattaniensis* differs from *Dulichiella cuvettensis*, *Dulichiella guinea* and *Dulichiella lecroyae* by having 2 accessory spines on pereopods 3-4 dactyli vs. 1 accessory spine. A summary of these distinguishing characters are given in Table 1.

**Table 1.** A summary of the diagnostic characteristicsthat serve to distinguishclosely related *Dulichiella* species.

	accessory flagellum	lateral cephalic lobe	male large G2 distolateral crown	male G1 coxa anterior margin	male G1 carpus: propodus	male G2 dactylus	pereopod 3–4 dactyli	pereopod 6-7	pleonite/urosome formular	epimera 3 posteroventral margin
*Dulichiella pattaniensis*	5 articles	truncate	with 4 spines, fourth spine not well developed	straight	>	overlapping into palm posterodistal corner	with 2 accessory spines	with bunch of long setae on merus carpus and propodus	7-7-7-5-6-2	smooth
*Dulichiella appendiculata*	5 articles	truncate	with 4 spines, fourth spine not well developed	concave	=	fitting into palm posterodistal corner	with 2 accessory spines	with bunch of long setae on basis merus carpus and propodus	7-7-7-5-4-2	smooth
*Dulichiella cotesi*	3 articles	truncate	with 3 spines	straight	=	fitting into palm posterodistal corner	with 1 accessory spine	with bunch of long setae on merus carpus and propodus	7-7-7-5-6-2	smooth
*Dulichiella cuvettensis*	4 articles	truncate	with 4 spines, fourth spine not well developed	straight	>	overlapping into palm posterodistal corner	with 1 accessory spine	with bunch of long setae on carpus and prooodus	7-7-7-5-4-2	serrate
*Dulichiella fresnellii*	4 articles	truncate	with 4 spines, fourth spine not well developed	concave	>	overlapping into palm posterodistal corner	with 2 accessory spines	with bunch of long setae on carpus and prooodus	7-7-7-5-4-2	smooth
*Dulichiella guinea*	5 articles	truncate	with 4 spines, fourth spine well developed	straight	=	fitting into palm posterodistal corner	with 1 accessory spine	with bunch of long setae on basis merus carpus and propodus	7-7-7-5-4-2	smooth
*Dulichiella lecroyae*	4 articles	rounded	with 4 spines, fourth spine well developed	straight	>	fitting into palm posterodistal corner	with 1 accessory spine	with bunch of long setae on carpus and prooodus	7-7-7-5-4-2	smooth
*Dulichiella oahu*	4 articles	truncate	with 3 spines	straight	=	fitting into palm posterodistal corner	with 2 accessory spines	with bunch of long setae on carpus and prooodus	7-7-7-5-6-2	smooth
*Dulichiella pacifica*	4 articles	truncate	with 4 spines, fourth spine well developed	straight	=	fitting into palm posterodistal corner	with 1 accessory spine	with bunch of long setae on carpus and prooodus	7-7-7-5-4-2/6-2	smooth
*Dulichiella takedai*	4 articles	truncate	with 4 spines, fourth spine well developed	straight	>	overlapping into palm posterodistal corner	with 2 accessory spines	with bunch of long setae on merus carpus and propodus	7-6-7-5-4-2	serrate
*Dulichiella tulear*	no data	truncate	with 3 spines	convex	<	fitting into palm posterodistal corner	with 1 accessory spine	without bunch of long setae	7-7-7-5-6-2	serrate

#### *Melita* Leach, 1841

##### Melita latiflagella

Ren & Andress, 2012

http://species-id.net/wiki/Melita_latiflagella

Figures 10 , 11

###### Material examined.

Lower Gulf of Thailand, Songkhla Lake (09°18'39.5"N, 99°46'46.4"E), 1 Feb 2012, Wongkamhaeng, K. PSUZC-CR-0191. (10♂; 10♀).

**Figure 10.**
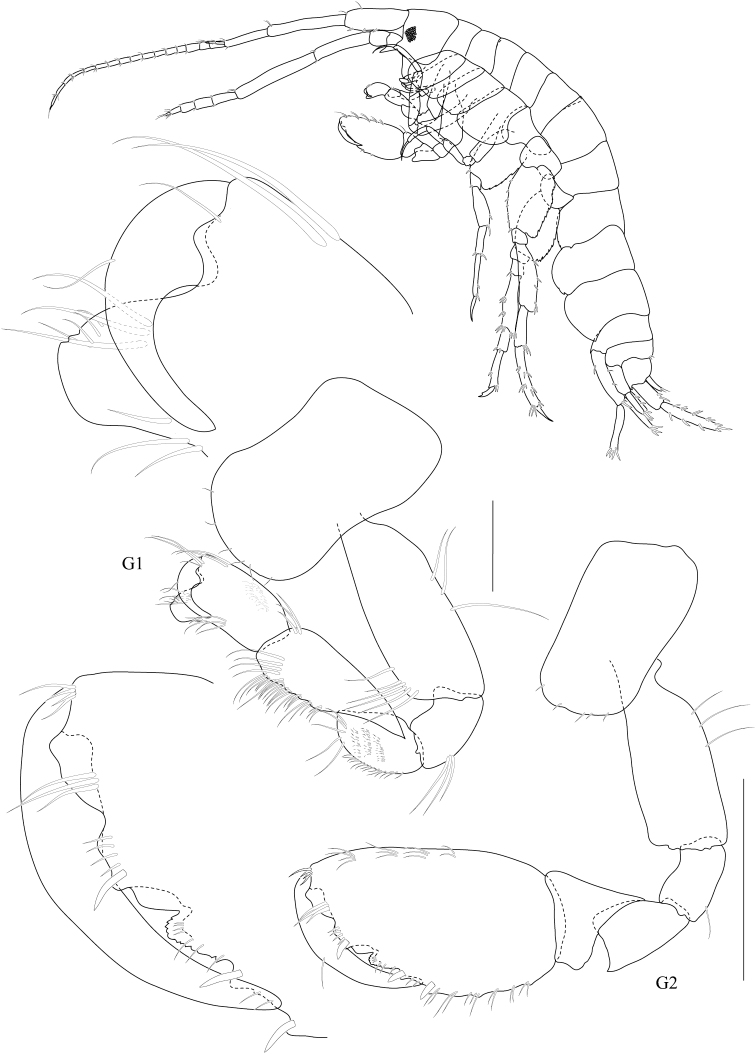
*Melita latiflagella* male (PSUZC-CR-000191) 4 mm. Outer Sonkhla Lake, lower Gulf of Thailand. All scale bars represent 0.2 mm.

**Figure 11.**
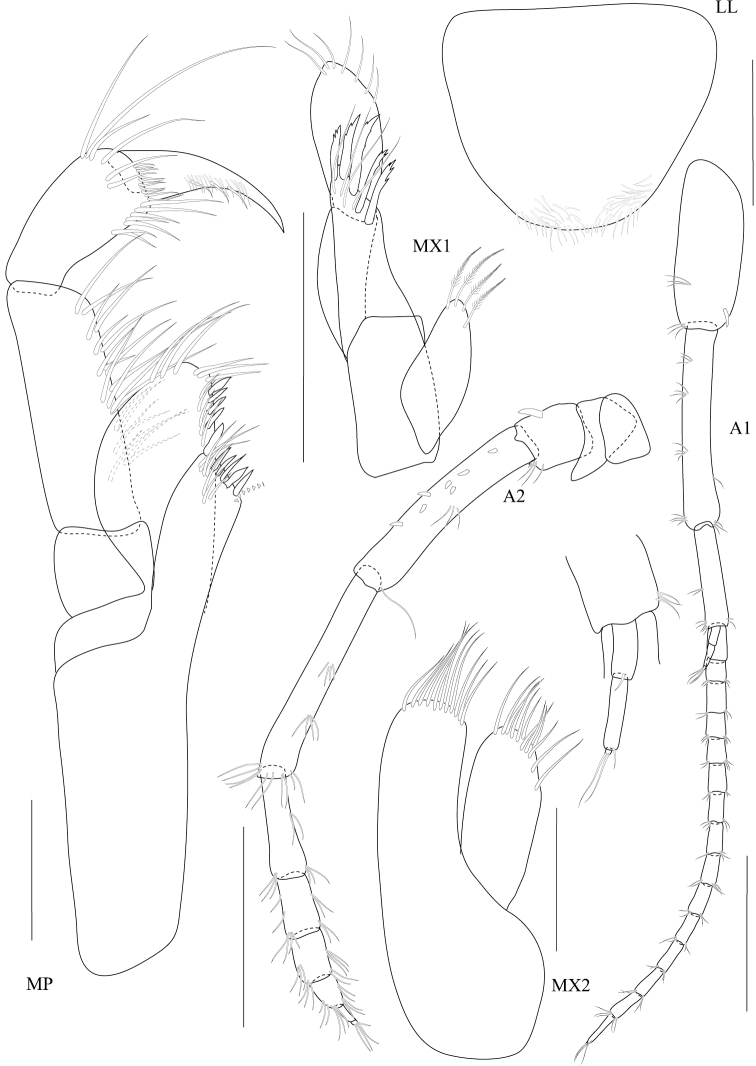
*Melita latiflagella* male (PSUZC-CR-000191) 4 mm. Outer Sonkhla Lake, lower Gulf of Thailand. All scale bars represent 0.2 mm.

###### Type locality.

Hainan province, China Sea.

###### Description.

*Head*. Lateral cephalic lobe smooth. *Antenna 1* peduncular article 1 longer than article 2, posterior margin with 2 marginal robust setae and 1 ventrodistal robust seta; flagellum with 19 articles, accessory flagellum 2 articles. *Antenna 2* gland cone not reaching to the end of article 3; flagellum with 5 articles. *Lower lip* inner lobes well developed, outer lobes pubescent. *Maxilla 1* inner plate with 3 terminal plumose setae. *Mandibular palp* article 2 subequal to article 1.

**Pereon.** *Gnathopod 1* coxa anteroventral corner slightly produced, posteroventral corner expanded; merus-propodussetose; carpus longer than propodus; propodus transverse, venterodistal corner produced, without defining robust seta on anteroventral corner; dactylus overlapping palm. *Gnathopods 2* merus posterodistal corner produced; carpus naked; propodus 3 × of carpus length, palmar margin oblique, serrated, longer than hind margin, with 2 robust setae, posterodistal corner produced with a robust seta; dactylus fit with palmar margin. *Pereopod 3* coxa subrectangular. *Pereopod 4* similar to pereopod 3; coxa distally expanded. *Pereopod 5 and 6* basis posterior margin rounded. *Pereopod 7* basis posterior margin straight.

**Pleon.** *Epimera 1–3* rounded. *Pleonite 1–3* dorsally smooth. *Uropod 1* peduncle with venterodistal spine, bearing marginal robust setae, both rami with a row of marginal robust setae. *Uropod 2* peduncle shorter than rami; rami subequal. *Telson* cleft each half with 2 apical robust setae.

###### Remarks.

Ren and Andres (2012) described the China Sea residing *Melita latiflagella* as having an antenna 2 that is long and extended. The specimens from this study are similar to those of Ren’s, but smaller in size with a total length of 3 mm as opposed to 5 mm.

###### Distribution.

China Sea and Songkhla Lake (current study).

## Supplementary Material

XML Treatment for Dulichiella

XML Treatment for Dulichiella pattaniensis

XML Treatment for Melita latiflagella
